# Complete Genome Sequence of Mycoplasma felis Strain Myco-2, Isolated from an Equine Tracheal Wash Sample in Japan

**DOI:** 10.1128/MRA.00057-20

**Published:** 2020-02-27

**Authors:** Yuta Kinoshita, Hidekazu Niwa, Eri Uchida-Fujii, Toshio Nukada

**Affiliations:** a Microbiology Division, Equine Research Institute, Japan Racing Association, Shimotsuke, Tochigi, Japan; Broad Institute

## Abstract

Mycoplasma felis causes conjunctivitis in cats and respiratory diseases in horses. We report here the complete genome assembly of equine Mycoplasma felis strain Myco-2, which was isolated from an ill horse in Japan.

## ANNOUNCEMENT

Mycoplasma diseases are relatively uncommon in horse populations, but Mycoplasma felis is known to cause equine respiratory diseases. M. felis has been isolated from equine clinical cases of pleuritis (1, 2) and an outbreak case of lower respiratory tract disease (3). Its pathogenicity was confirmed by experimental infection; a pony that was inoculated in the thoracic cavity with a pure culture of *M. felis* showed signs of pleuritis (4). Analysis of the equine *M. felis* strain could help to reveal the genetic background in cases of equine mycoplasma disease.

A *Mycoplasma* strain was isolated from a tracheal wash sample of an ill horse suffering from a cough in 2010 in Japan. An aliquot of tracheal wash sample (300 μl) was directly inoculated into *Mycoplasma* NK medium (Miyarisan Pharmaceutical, Tokyo, Japan) and incubated at 37°C. After the color of the medium was confirmed to change from red to yellow, 10 μl of suspension was plated onto *Mycoplasma* NK agar medium (Miyarisan Pharmaceutical) and incubated at 37°C in 5% CO_2_. A single colony was picked and stored at −80°C until the following steps took place. The bacteria were incubated in *Mycoplasma* broth (Oxoid CM0403; Thermo Fisher Scientific, Tokyo, Japan) supplemented with *Mycoplasma* selective supplement G (Oxoid SR0059; Thermo Fisher Scientific). Phenol-chloroform DNA extraction was conducted in accordance with a previous method (5). A library for an Ion Torrent Personal Genome Machine (PGM; Thermo Fisher Scientific) was prepared using an Ion PGM Hi-Q View Chef reagent, and the library was sequenced with the Ion 316 chip v. 2 BC.

Short reads generated by Ion PGM were trimmed in Sickle v. 1.33 software (6) using a quality threshold of 27 and a length threshold of 100 bp. The same genomic DNA was also used for nanopore sequencing, and a library was prepared with a ligation sequencing kit (SQK-LSK109; Oxford Nanopore Technologies [ONT]) and a native barcoding expansion kit (EXP-NBD104; ONT). The library was sequenced on a MinION sequencer using a FLO-MIN106 R9.4 flow cell (ONT) in the fast base-calling mode of MinKNOW v. 3.3.2 software. Only the reads stored in the “pass” folder were used in the following steps. Long reads were trimmed in NanoFilt v. 2.5.0 software (7) using a quality threshold of 10, a length threshold of 1,000 bp, and trimming of 100 bp from both the start and the end of each read. We used 778,445 high-quality short reads with an average read length of 182.9 bp and 59,437 high-quality long reads with an average read length of 8.47 kb for hybrid genome assembly. *De novo* genome assembly was carried out in Unicycler v. 0.4.8 software (8) in “conservative” mode with the depth filter set to 0.5, and the assembled genome was annotated in DFAST v. 1.2.4 software (9). The complete genome consists of 841,695 bp (GC content, 68.9%), 734 coding sequences (CDSs), 30 tRNAs, and 9 rRNAs. The strain Myco-2 was subsequently identified as *M. felis*, with 98.2% average nucleotide identity to *M. felis* reference strain ATCC 23391.

### Data availability.

These data are deposited in DDBJ/ENA/GenBank under accession number AP022325. The raw reads are available under BioSample accession number SAMD00199257.
